# Wilfred Leslie Sleight

**Published:** 1935

**Authors:** 


### WILFRED LESLIE SLEIGHT, M.R.C.S., L.R.C.P.

It is with great regret that we have heard of the death, at the age of thirty-three, of Wilfred L. Sleight, who was killed by a blow from an aeroplane propeller in Australia. He was at the time employed as a medical officer in the P. & O. Company. He was a student in the University of Bristol and at the Royal Infirmary. After obtaining his medical qualification he was appointed House Physician, and subsequently Resident Obstetric Officer, at the Royal Infirmary. He proved himself to be an exceptionally good Resident, and promised to make a very good doctor. Sleight was deeply interested in the study of medicine, though much hampered by a total inability to express himself : and when he was given increasing responsibilities as a resident medical officer he gave undoubted proof of his real grasp of the subject of his studies, and of his capacity for action.

Mr. Gordon Paul, who was Senior Resident Officer at the Bristol Royal Infirmary, writes : "Sleight was a splendid resident, thoroughly reliable, diligent and painstaking, and with a sense of duty developed to a remarkable degree. For example, while he was the Obstetric House Surgeon, I never knew him absent himself from the Bristol Royal Infirmary without leaving his telephone number or instructions as to where he might be found. As a man he was universally popular amongst his colleagues. I cannot recall his ever having said a harsh word against anyone, and one always felt instinctively that one could entrust him with anything, knowing that it would be done—and well done. His loss is a severe blow to his friends, and his place will not be readily filled. Although we shall see him no more, he leaves behind him the memory of a delightful personality and staunch friend."

To his sorrowing parents we extend our deepest sympathy in their terrible loss.

---

### Meetings of Societies

